# Contradictions and convergences in recommendations on physical activity in pregnancy in different countries after the publication of the WHO guidelines in 2020—a scoping review

**DOI:** 10.3389/fpubh.2025.1540355

**Published:** 2025-04-28

**Authors:** Aneta Worska, Janusz Maciaszek, Julia Ciążyńska, Anna Szumilewicz

**Affiliations:** ^1^Department of Physical Activity and Health Promotion Science, Poznan University of Physical Education, Poznan, Poland; ^2^Department of Fitness, Gdansk University of Physical Education and Sport, Gdansk, Poland

**Keywords:** guidelines, recommendations, physical activity, exercise, pregnancy

## Abstract

**Objectives:**

The main objective of this review is to determine whether the physical activity (PA) recommendations during pregnancy issued by public health and sports medicine organizations published since 2020 in different countries around the world converge or differ and what are the emerging trends in these guidelines.

**Methods:**

The review was conducted as per the PRISMA Extension for Scoping Reviews (PRISMA-ScR). We searched scientific databases (PubMed, ScienceDirect, Web of Science, Academic Search Complete, and SPORTDiscus with Full Text via EBSCO) and the Internet to identify papers regarding official recommendations on PA during pregnancy published by public health and sports medicine organizations. We analyzed 10 eligible guidelines, published from 2020 in English from eight countries and two international organizations.

**Results:**

The analysis of the guidelines revealed that all of them advocate for moderate-intensity PA during pregnancy. Seven documents recommended also vigorous or high-intensity activities. Some guidelines recommend it only after consultation with a healthcare provider, particularly for participants with specific health conditions. The analysis of the guidelines shows a convergence on the frequency and duration of PA, suggesting at least 150 min per week.

**Conclusion:**

There is a general convergence around the safety and benefits of moderate-intensity PA during pregnancy. There is a divergence in recommendations regarding higher-intensity exercise and altitude training, with limited specific guidance for these activities. However, we have seen much greater openness in this area over recent years. Our work highlights a knowledge gap regarding the safety and efficacy of more intense exercise regimens for pregnant women, emphasizing the need for further research to develop evidence-based guidelines and to address new trends in pregnant population.

**Systematic review registration:**

The study protocol was registered on Open Science Framework (registration number: 10.17605/OSF.IO/3QFR9).

## 1 Introduction

Physical activity (PA) is widely recognized as an important component of a healthy lifestyle, offering various benefits for both physical and mental well-being (1). The importance of PA during pregnancy is increasingly appreciated, as it has been shown to affect both maternal and fetal health (2, 3), as well as potentially reducing the risk of certain pregnancy-related complications (4). Despite this evidence, pregnant women often reduce their PA levels during pregnancy (5). Studies indicate that pregnant women engage in the highest level of PA in the second trimester, and gradually reduce their activity as the pregnancy progresses (6, 7). Studies have shown that pregnant women face challenges in meeting PA recommendations due to various barriers. These barriers include physiological and anatomical changes, socio-cultural factors, lack of clear exercise guidelines (8), and lack of information and social support (9).

Public health and sports medicine organizations must adhere to specific guidelines when making official recommendations to help ensure the quality and reliability of the information provided. These guidelines are crucial for maintaining consistency, evidence-based practices, and ethical standards in public health decision-making. The World Health Organization (WHO) has a vital role in providing advice and guidance for public health actions. The WHO’s guidelines are a useful tool for countries around the world to develop and implement public health policies (10, 11). The WHO “Who Guidelines On Physical Activity And Sedentary Behavior” (12) explains how the WHO customizes guidelines for various regions with scientific rigor and local context in mind. General reference to the principles of evidence-based healthcare, alongside a clear framework for the creation and dissemination of guidelines, can be found in “Building Capacity for Evidence-Based Public Health: Reconciling the Pulls of Practice and the Push of Research” (13) which discusses the importance of evidence-based practices in public health.

Increasingly, experts recognize the value of teamwork between healthcare providers and exercise professionals, especially when encouraging pregnant women to be physically active. This collaborative approach, with the woman at the center, is emphasized in research as crucial for optimal health outcomes (14). In 2016, EuropeActive established guidelines for exercise professionals working with pregnant women to elevate the standards of prenatal and postnatal PA (15). The standards were revised in 2022 to reflect current trends in pregnancy exercise (16). This document was the initial one to outline the required learning outcomes for creating and executing a prenatal workout plan according to the European Qualifications Framework. By setting standards for exercise professionals working with pregnant and postpartum women, these guidelines help ensure that exercise programs are both safe and effective. This standardized training helps guarantee that exercise specialists are prepared to implement official recommendations for exercise during pregnancy and after childbirth. Pregnancy and postpartum exercise professionals increase their professional credibility with pregnant women and healthcare providers by following these guidelines to effectively assist and guide clients with tailored exercise routines for pregnancy and postpartum periods.

Considering the multiple benefits of PA, including during pregnancy, it is crucial to understand and adhere to appropriate guidelines that consider the individual’s needs and capabilities. Various organizations and research studies have provided recommendations for PA during pregnancy, but there is still a lack of consensus on the specific guidelines to follow (17). This lack of consensus creates a challenge for pregnant women in determining the appropriate level and type of PA to engage in during pregnancy (18). In 2015, in our previous review study (19) we showed that guidelines provide limited information on the specifics of prenatal exercise content and how to adapt sports activities to pregnancy. As a result, we concluded that guidelines should be updated based on high-quality research and in collaboration with practitioners to be more comprehensive and actionable. In a follow-up review conducted in 2021, we showed that there was still a need for updated guidelines that reflect current high-quality research and practical experience (20). An important statement in the WHO guidelines published in 2020 was that “Women who, before pregnancy, habitually engaged in vigorous intensity aerobic activity, or who were physically active, can continue these activities during pregnancy and the postpartum period.” On the one hand, this reflected the greater body of scientific evidence regarding higher intensity PA in pregnancy. On the other hand, it may have been a response to the social needs of pregnant recreational and elite athletes who did not want to reduce the intensity of PA after becoming pregnant. In this review, we wanted to consider new trends emerging in official guidelines for participation in PA during pregnancy.

PA is defined as “all bodily actions produced by the contraction of skeletal muscle that increase energy expenditure above basal level” (21). This includes a wide range of movements and activities that people engage in every day. PA can be categorized in various ways, including by its intensity (e.g., light, moderate, vigorous, high), type (e.g., aerobic, strength-conditioning, balance, stretching exercises), and purpose (occupation, exercise, household chores, recreation, sports) (22). Although PA recommendations should always be adapted to the individual needs and capabilities of the participants, there are common components characterizing PA: frequency (how many exercise sessions per week?), time (how long is each exercise session?), type (type of exercise), and intensity (how hard or difficult is the exercise?). These elements are crucial in developing effective PA guidelines for pregnant women to ensure optimal health outcomes for both the mother and the fetus (18).

National and international health organizations, including the WHO, advocate for regular PA during pregnancy, emphasizing benefits for both mother and child (12, 20). However, a critical distinction often overlooked is the difference between promoting general PA and prescribing specific exercise programs. The WHO guidelines, for instance, broadly encourage PA, including activities such as walking, housework, and occupational activity (12, 19, 20), primarily focusing on reducing sedentary behavior and increasing overall movement. This approach aligns with a public health perspective aimed at improving population-level health outcomes. However, this broad recommendation may not adequately address the needs of pregnant women seeking guidance on structured exercise programs (14). This gap necessitates exploring the nuances of purpose, type, intensity, frequency, and duration, particularly concerning *structured exercise programs* designed for specific fitness goals (20). The following review addresses these different approaches, analyzing recommendations for both general PA and structured exercise programs and highlighting the need for further research to optimize exercise guidance during pregnancy. The aim of this study was to determine whether there is convergence or divergence in the recommendations regarding PA during pregnancy issued by public health and sports medicine organizations since 2020 and what are the emerging trends in these guidelines. To achieve this, we will analyze the details of these recommendations, specifically examining areas of agreement or disagreement regarding frequency, time/duration, type, intensity, monitoring methods of PA.

## 2 Materials and methods

### 2.1 Identifying the research question

In our study, we identified two questions:

1. Is there convergence between the PA recommendations for pregnant women issued since 2020 by public health and sports medicine organizations in terms of frequency, time/duration, type, intensity, methods of its monitoring?
2. Is there contradiction between the PA recommendations for pregnant women issued since 2020 by public health and sports medicine organizations in terms of frequency, time/duration, type, intensity, methods of its monitoring?

### 2.2 Inclusion and exclusion criteria

**Figure 1:**
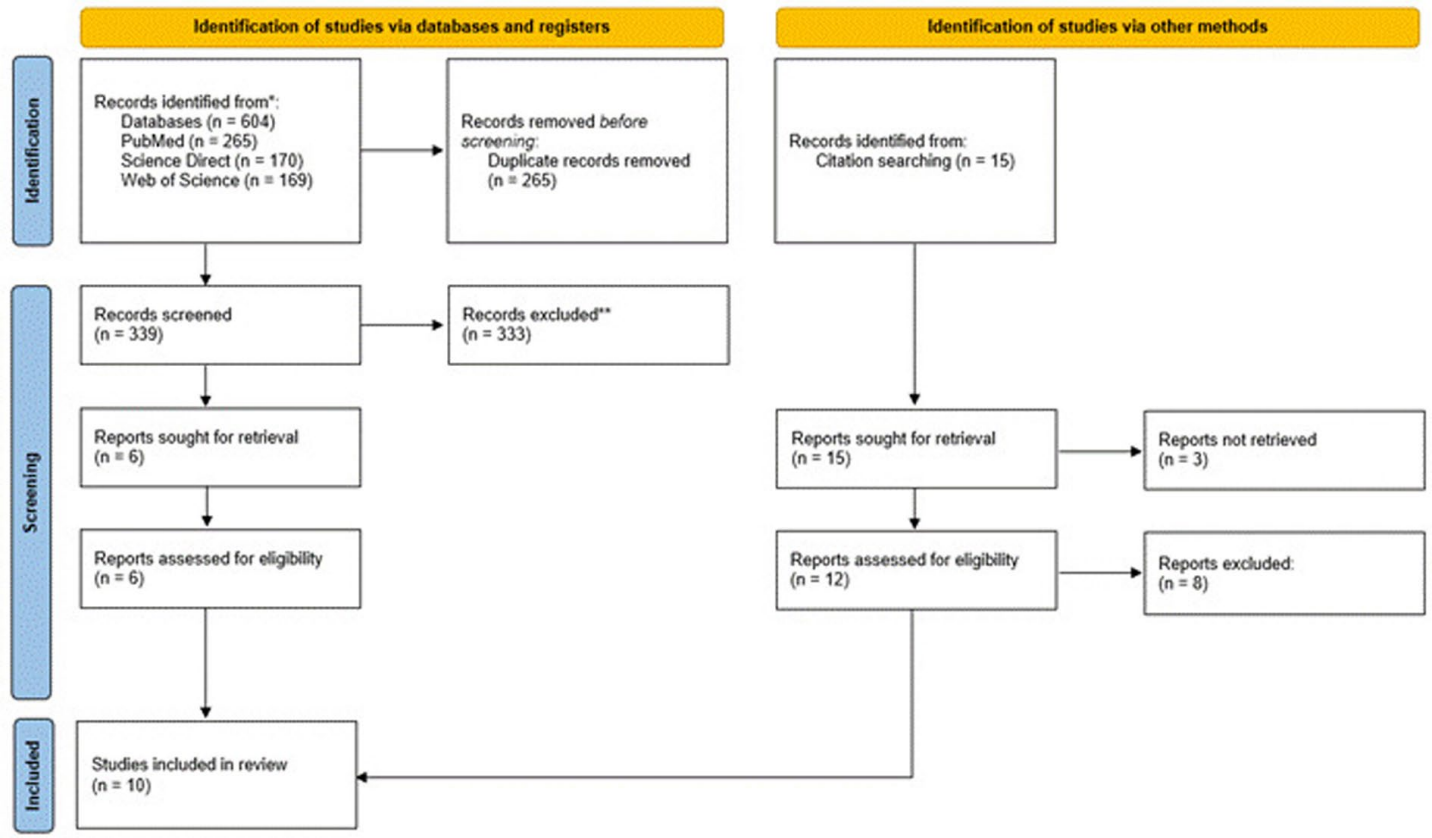
Overview of the screening and paper selection process (PRISMA flowchart).

The review was conducted according to the PRISMA Extension for Scoping Reviews (PRISMA-ScR) (23) in February 2024. The study protocol was registered on Open Science Framework (registration number: 10.17605/OSF.IO/3QFR9). Figure 1 shows the PRISMA diagram of the article screening process (24). Studies from January 2020 to February 2024 were considered for inclusion based on inclusion/exclusion criteria (Table 1). The review included officially recognized guidelines for PA during pregnancy, targeted at healthy pregnant women, published in English (or with available translations) on or after the publication of the 2020 WHO guidelines. The guidelines needed to focus on general PA or structured exercise programs (recommendations for PA type, intensity, duration, and frequency). We excluded non-guideline documents, guidelines for pregnant women with specific health conditions (unless addressed within broader guidelines), pre-2020 guidelines (unless significantly updated), and non-English guidelines without translations.

**Table 1:** Inclusion/Exclusion criteria for the selection of paper.

Inclusion	Exclusion
Published in English	Other language than English
Published by Public Health or Sports Medicine Organizations	Other than Public Health or Sports Medicine Organizations papers
Published since 2020	Reviews, editorials, commentaries, and qualitative studies

### 2.3 Search strategy

A systematic search for guidelines and recommendations on PA during pregnancy was conducted in February 2024. PubMed, ScienceDirect, and Web of Science databases were searched. Search string: “(“pregnancy” AND (“physical activity” OR “exercise”)) AND (“recommendations” OR “guidelines”) AND (“public health” OR “sports medicine organization”).” Guidelines issued by national representative bodies, which do not necessarily constitute peer-reviewed research publications, were collected in a secondary search of publicly available online resources using the previously described search string. The documents could have been published in 2020 (the year of publication the recent WHO guidelines) and later. The search omitted non-peer-reviewed documents.

### 2.4 Selection of studies

From the search results, duplicates were removed. Two authors screened study titles using the inclusion/exclusion criteria (Table 1). Studies with titles implying eligibility for inclusion had the abstract screened. If the abstract indicated the study may be eligible for inclusion, then the full text was downloaded for review. The references of papers were analyzed to identify additional papers fulfilling the inclusion criteria that may have been missed by the search strategy. The full texts were procured and independently reviewed and analyzed by two investigators (AW and JC) using inclusion/exclusion criteria. In the event of a split opinion as to whether a particular document should be qualified for examination, a third investigator (JM) was ready to make the final decision.

### 2.5 Data charting

10 papers were selected for data charting (25–34). The data from selected articles were extracted independently in a Microsoft Excel sheet by three investigators (AW, AS, JC) and put into a form designed after deliberations by all the authors (Table 2). The following data were charted: authors or publishing organizations, country, year of publication, and title. Next we extracted information related to PA during pregnancy based on FITT principle: recommended type of PA (divided into two subsections: recommendation and explanation/examples), intensity of PA (divided into two subsections: recommendation and explanation/examples), methods of monitoring the PA intensity (divided into two subsections: recommendation and explanation/examples), recommended time/duration of PA, recommended frequency of PA, information on what is not recommended in relation to PA during pregnancy, other information in relation to PA, consistency of the information included. In the category “other information in relation to PA” we treated information related to warning signs, contraindications, bed rest, elite athletes, and environment (high altitude, high temperature, humidity, etc.). In the following text, we use the term “PA information categories” to refer to information obtained based on the extraction criteria mentioned above.

**Table 2:** Short characteristics of analyzed guidelines on physical activity published since 2020.

Autor or publishing organizations	Country	Year of publication	Type of physical activity		Intensity of physical activity		Methods of monitoring the physical activity intensity		Time/duration of physical activity	Frequency of physical activity	Information on what is not recommended in relation to physical activity during pregnancy	Other information in relation to physical activity	Consistency of the information contained in the document
			Recommendation	Explanation/examples	Recommendation	Explanation/examples	Recommendation	Explanation/examples	Recommendation	Recommendation			
American College of Obstetricians and Gynecologists (ACOG) (26)	United States	2020	Aerobic exercises Strength-conditioning exercises Stretching exercise Water exercises	Included	Moderate Higher under strict medical supervision Obesity: low to comfortable	Included	Borg ratings of perceived exertion scale The 'Talk Test'	Included	30–60 minutes	At least 3–4 times per week (up to daily)	Included	Included	Yes
Austrian Health Promotion Fund (27)	Austria	2020	Aerobic activity Strength-conditioning exercises Pelvic floor training	Included	Moderate	Not included	Not included	Not included	At least 150 minutes per week	Muscle-strengthening exercises: recommended on 2 or more days a week, involving all major muscle groups	Included	Included	Yes
British Association of Sport and Exercise Medicine (28)	United Kingdon	2020	Aerobic exercise Strength conditioning exercises	Included	Moderate	Included	Not included	Not included	At least 150 minutes per week	Strength conditioning exercises twice a week	Included	Included	Yes
Department of Health Australian Government (29)	Australia	2020	Aerobic exercises Strength conditioning exercises Pelvic floor exercise	Included	Moderate to vigorous Higher after consulting a health professional	Not included	Rating of Perceived Exertion The 'Talk Test'	Included	150 to 300 minutes (2½ to 5 hours) of moderate intensity physical activity 75 to 150 minutes (1¼ to 2½ hours) of vigorous physical activity An equivalent combination of both moderate and vigorous activities, each week	Muscle strengthening activities on at least 2 days each week	Included	Included	Yes
World Health Organization WHO (30)	International	2020	Aerobic exercises Strength conditioning exercises Gentle stretching Pelvic floor exercise	Not included	Moderate Vigorous, if before pregnancy, habitually engaged in vigorous intensity aerobic activity, or who were physically active	Not included	Not included	Not included	At least 150 minute per week Pregnant women should start by doing small amounts of physical activity, and gradually increase frequency, intensity and duration over time.	Pelvic floor muscle training may be performed on a daily basis Pregnant women should start by doing small amounts of physical activity, and gradually increase frequency, intensity and duration over time.	Included	Included	Yes
Australian Government. Department of Health (AGDH) (31)	Australia	2021	Aerobic exercises Strength conditioning exercises Pelvic floor exercise	Included	Moderate to vigorous	Not included	Tthe ‘Talk Test’	Included	Moderate intensity activities for 2½ to 5 hours each week Vigorous intensity activities for 1¼ to 2½ hours each week Muscle strengthening activities at least 2 days each week	Muscle strengthening activities at least 2 days each week	Included	Included	Yes
Department of Ergometry, Exercise, Nuclear Cardiology, and Cardiovascular Rehabilitation of the Brazilian Society of Cardiology (32)	Brasil	2021	Aerobic exercises (low impact) Strength conditioning exercises Stretching exercisePelvic floor muscle exercises	Included	Aerobic: Moderate Resistance exercise: Moderate	Included	The perceived exertion with a Borg scale The 'Talk Test'	Included	Minimum of 150 min of moderate-intensity exercise/week Aerobic: 20 to 30 minutes per session (goal - a total of 150 minutes/week) 10 to 15 minutes warm-up and cool-down Resistance: 15 to 20 minutes 10 to 15 minutes of warm-up and cool-down Swimming/aqua aerobic: up to 45 minutes/session	3 to 5 days/week	Not included	Included	Yes
European Board and College of Obstetrics and Gynaecology (EBCOG) (33)	International	2023	Aerobic exercises Strength conditioning exercises	Included	Moderate to intense High with caution and can continue if previously habitually engage	Included	Not included	Not included	At least 150 minutes per week	Not included	Included in the context of the need for caution	Not included	Yes
Polish Society of Gynecologists and Obstetricians (PTGiP) and Polish Society of Sports Medicine (PTMS) (34)	Poland	2023	Endurance exercise Resistance exercises Stretching exercises Neuromotor exercise Pelvic floor exercise Individualized	Included	Moderate to high Individualized Resistance exercise: until moderate fatigue Competitive athlete: > 90% of maximum exercise capacity is safe (in any trimester)	Included	Heart Rate response to exercise and/or ratings of perceived exertion Ratings of Perceived Exertion scale (RPE), eg Borg scale The "Talk Test"	Included	At least 30 min of moderate-intensity exercise per day, up to a total of at least 150 minutes per week 75 minutes of high-intensity exercise per week Suggestions for training volume adjustment in pregnancy; • first trimester = up to 80% of the pre-pregnancy values; • second trimester = up to 90% of the pre-pregnancy values; • third trimester = up to 50% of the pre-pregnancy values.	Resistance: 2–3 non-consecutive days per week Stretching/neuromotor: at least 2–3 to 7 days per week Pelvic floor: 1 to 7 days per week	Included	Included	Yes
Royal Australian and New Zealand College of Obstetricians and Gynaecologists (RANZCOG) (35)	Australia & New Zealand	2023	Aerobic exercises Strength conditioning exercises Pelvic floor exercise	Included	Moderate to vigorous	Included	The Heart Rate response to exercise and/or ratings of perceived exertion Pregnancy-specific Heart Rate zones equivalent to 60-80% of maximal aerobic capacity The Rating of Perceived Exertion scale (RPE) The “Talk Test”	Included	150 to 300 minutes of moderate intensity physical activity each week (most days of the week for at least 30 minutes at a time) For previously inactive women and overweight or obese, a shorter duration of exercise (15-20 minutes) before slowly building up to 30 minut	Most, preferably all days of the week Two sessions of strengthening exercises per week, on non-consecutive days	Included	Included	Yes

## 3 Results

### 3.1 Study characteristics

A systematic search of public health and sports medicine organizations` guidelines for pregnancy identified a total of 619 papers for evaluation. After further exclusion (the rejection criteria are included in Table 1) and removing duplicates and papers irrelevant to the selected topic, 6 papers were selected for data charting (25, 29, 31–34). Based on the reference lists presented in these papers, an additional 4 studies were included (26–28, 30). Two recommendations were published in Australia, one each in Australia and New Zealand, Austria, Brazil, Poland, the United Kingdom, and the United States, and two by International Organizations.

### 3.2 Outcomes

Ten documents containing recommendations on PA during pregnancy from eight countries were analyzed: Australia, Australia & New Zealand, Austria, Brazil, Poland, the United Kingdom, the United States, and two international organizations: European Board and College of Obstetrics and Gynecology (EBCOG) and World Health Organization (WHO) looking for information on recommendations related to PA core components and other additional information’s related to them (Table 2).

#### 3.2.1 Recommendations for type of PA

Analysis of the documents showed a convergent recommendation for the type of PA. The most commonly recommended types of PA based on the documents provided are aerobic exercises (such as walking, running, cycling, swimming, and other forms of aerobic exercises that increase heart rate and improve cardiovascular health) and strength-conditioning exercises (include exercises aimed at increasing muscle strength and endurance, such as weight lifting, resistance training, and bodyweight exercises) recommended by all documents, and pelvic floor exercises (targeted exercises to strengthen the pelvic floor muscles,) recommended in six documents (26, 28–30, 33, 34). Three papers additionally recommended stretching (29, 32, 33), and only one recommended neuromotor exercises (33). One document recommended specific type of PA (aerobic and muscle-strengthening activities, gentle stretching, pelvic floor), but did not explain/exemplify what forms of PA can be understood by them (29).

#### 3.2.2 Recommendations for intensity of PA

All organizations commonly recommend moderate-intensity PA. It is often defined as activity that increases the heart and breathing rates to a level where the individual can still talk but not sing during the activity. It is equivalent to about 3.0 to less than 6.0 metabolic equivalents. Three documents do not explain what moderate PA is (26, 28, 29). Six documents also include information on vigorous or high-intensity activities (25, 28, 30, 32–34). No document explicitly discourage high-intensity PA. However some documents recommend higher intensity ‘under strict medical supervision’. Three documents recommend higher intensity after consulting the doctor (25, 28, 32), especially for individuals with specific health conditions such as obesity (25). Four documents stated that women who were active before pregnancy, can continue their usual PA behavior and sports activities, as long as they feel comfortable (25, 29, 32, 33). One document additionally states that “according to clinical data, a pregnant competitive athlete’s training intensity (in any trimester) that does not exceed 90% of her maximum exercise capacity during pregnancy is safe for the mother and fetus.” (33). For the first time the Polish guidelines discussed the effects of high intensity interval training (HIIT) performed during pregnancy.

#### 3.2.3 Recommendations for methods of monitoring the PA intensity

Six documents include information on intensity monitoring methods (25, 28, 30, 31, 33, 34). These organizations, similarly suggest using the Borg ratings of perceived exertion scale or the “Talk Test” as methods for monitoring exercise intensity. Two additionally cite individual heart rate ranges (33, 34). Only five of them contain additional information, explaining what these methods are and how to use them (25, 28, 30, 31, 33, 34).

#### 3.2.4 Recommendations for time/duration and frequency of PA

Recommendations for the duration of a single training unit and the total weekly time devoted to PA are consistent. With reference to single training unit duration The American College of Obstetricians and Gynecologists (25) recommend a duration of 30–60 min per exercise session at least 3–4 times per week (up to daily). All organizations take the same stance, recommending at least 150 min a week. The Department of Health Australian Government (28) recommends a range of 150 to 300 min of moderate-intensity PA or 75 to 150 min of vigorous-intensity PA each week, or an equivalent combination of both moderate and vigorous activities. Likewise Polish Society of Gynecologists and Obstetricians (PTGiP) and Polish Society of Sports Medicine (PTMS) (33) and Royal Australian and New Zealand College of Obstetricians and Gynecologists (RANZCOG) (34).

#### 3.2.5 Information on what is not recommended in relation to PA

The documents analyzed unanimously indicate the need for pregnant women to avoid contact activities with high risk of abdominal trauma (25, 27–30, 32, 33) or loss of balance and falling (i.e., activities that require high levels of balance, coordination and agility) (25, 27–30, 32, 33) as well as those involving significant changes in oxygen partial pressure (e.g., sky diving, scuba diving) (25, 27–30, 32, 33). One document has not included information at all on what is discouraged/recommended to avoid during pregnancy in the context of PA (31).

WHO generally recommend avoiding PA at high altitudes, but do not specify exactly what altitude (29). Five other organizations (25, 27, 30, 32, 33) provide accurate metric, but here there is minor discrepancy. The American (25) and British (27) guidelines recommend caution above 6,000 feet (about 1,800 m), European Board and College of Obstetrics and Gynaecology (32) stated that ‘Country-specific guidelines recommend that exercise needs to be avoided at altitudes greater than 1800 m, at least until the body physiologically fully adjusts to the altitude’. Similarly the Australian guideline (30) recommend caution above 2,000 m ‘unless acclimatised and trained for the activity before pregnancy’. The Polish (33) one quotes the Canadian recommendation that ‘women living in lowland areas (i.e., below 2,500 m above sea level) should avoid PA at high altitudes (> 2,500 m above sea level)’. However, the Polish experts also underline that so far ‘there is no data on the safety of altitude training for competitive female athletes’.

#### 3.2.6 Other information in relation to PA

Eight guidelines provide converging advice on sedentary lifestyles (25–30, 33, 34), offering general advice such as ‘Women who have been sedentary before pregnancy are recommended to follow a gradual progression of exercise—‘not active—start gradually’ (27), or ‘Break up long periods of sitting as often as possible’ (28, 30). The same applies to recommendations for women who were active before pregnancy. Women who were active before pregnancy, can continue their usual PA behavior and sports activities, as long as they feel comfortable or are adapted to it (25–30, 32–34). Six guidelines list warning signs for stopping PA during pregnancy (25, 27, 28, 30, 33, 34). All are in line, with factors such as chest pain, persistent shortness of breath that does not get better with rest, severe headache, persistent dizziness/feeling faint that does not get better with rest, regular painful uterine contractions, vaginal bleeding, persistent loss of fluid from the vagina—indicating possible ruptured membranes. Seven documents provide recommendations for pregnant athletes (25, 28–31, 33, 34). They all unanimously recommend that athletes may continue with their PA/exercise program, but should modify their activities as their pregnancy progresses, with advice from an informed and appropriately qualified health professional.

#### 3.2.7 Clarity and comprehensibility of the recommendations

All recommendations seem clear and understandable. The vocabulary and the way they are written should be understandable to all, medical staff, health-promoting exercise professionals as well as pregnant women and others reading them. Each document appears consistent in relation to the information it contains. There are no internal inconsistencies. Moreover, they are also consistent between countries in most general recommendations, but divergence appears in the degree of detail and against some specific recommendations.

## 4 Discussion

### 4.1 About the findings

Based on the information provided, there are no explicit divergences in results evident from the analyzed documents regarding PA recommendations during pregnancy. The recommendations appear to be largely congruent, particularly emphasizing moderate-intensity PA, individualization based on the woman’s health and fitness level, and consultation with healthcare providers for those with any contraindications or pre-existing conditions. This aligns closely with the findings presented by Hayman et al. (35) in their scoping review, which highlights that guidelines from multiple countries recommend 150–300 min per week of moderate-intensity aerobic activity for women with uncomplicated pregnancies, alongside pelvic floor and muscle-strengthening exercises. Moreover, the authors underscores the global agreement on the necessity of adapting PA recommendations to individual circumstances.

Importantly, the recommendations provided by various health and sports medicine organizations are often detailed enough to help plan a safe and effective exercise session for pregnant women. Guidelines like those from the American College of Obstetricians and Gynecologists (25), Australian Government. Department of Health (AGDH) (30), British Association of Sport and Exercise Medicine (27), Department of Ergometry, Exercise, Nuclear Cardiology, and Cardiovascular Rehabilitation of the Brazilian Society of Cardiology (31), Polish Society of Gynecologists and Obstetricians (PTGiP) and Polish Society of Sports Medicine (PTMS) (33), and Australian and New Zealand College of Obstetricians and Gynaecologists (RANZCOG) (34) typically offer clear instructions on the types and intensity of exercise that are considered safe and beneficial during pregnancy. It seems very important that all countries can offer such guidelines in their national languages to promote PA during pregnancy worldwide.

Regular PA confers numerous health benefits for pregnant women, aligning with the 2020 World Health Organization’s guideline (29) emphasizing the importance of aerobic and muscle-strengthening activities during pregnancy. However, a crucial distinction exists between *general physical activity* and *structured exercise programs*, which influences the practical application of these recommendations. The WHO guideline (29), while advocating for at least 150 min of moderate-intensity aerobic activity per week, predominantly focus on promoting overall PA, encompassing any bodily movement like walking, housework, or occupational tasks. In contrast, *structured exercise programs* involve planned, repetitive, and purposeful PA with specific goals for improving or maintaining physical fitness (27) such as swimming, prenatal yoga, or supervised strength training (20).

This distinction is essential because pregnant women and healthcare providers often seek guidance on *structured exercise programs* rather than simply increasing general PA. While the WHO guideline (29) offer valuable public health advice, it lacks the specific exercise prescriptions needed to address the nuances of adapting exercise routines throughout pregnancy, including type, intensity, frequency, and duration. This gap is addressed by various national and professional bodies, such as those in Australia (30), Brazil (31), Poland (33), and New Zealand (34), which provide more detailed recommendations, often tailored to specific populations and circumstances. That guideline, while valuable for its practical advice, frequently lack the rigorous peer-review process of scientific publications, highlighting the need for further research to validate and refine these recommendations, especially in the context of high-intensity interval training and exercise at altitude. The analyzed documents lack detailed information on how to encourage women to engage in regular PA during pregnancy. It is evident that despite the known benefits of PA during pregnancy, a significant proportion of pregnant women do not meet the recommended guidelines (36). Studies have shown that pregnant women often reduce their PA levels during pregnancy, indicating a gap between expert recommendations and actual behavior (37). Furthermore, the lack of motivation, fear of potential fetal harm, and individual attitudes and practice habits of healthcare providers can influence the engagement of pregnant women in PA (38). Healthcare providers are often the primary source of PA information for pregnant women. Okafor and Goon (39) highlighted that effective counselling from healthcare professionals is associated with increased uptake of PA by African pregnant women. They found that women who received specific advice or counselling about PA were more likely to engage in and maintain regular PA. This aligns with findings from Sparks et al., who noted that adapted PA prescriptions from healthcare providers could enhance adherence to recommended guidelines (40).

Some contradictions between public health and sports medicine PA guidelines for pregnant women can stem from different objectives and interpretations of scientific evidence. Public health guidelines, such as those issued by the American College of Obstetricians and Gynecologists (25) typically emphasize the safety and well-being of the general population of pregnant women. They often recommend at least 150 min per week of moderate-intensity aerobic activity for healthy pregnant women, unless there are medical or obstetric complications. On the other hand, sports medicine organizations might provide more nuanced guidance for specific populations, such as athletes, and may support more vigorous exercise regimens as long as they are carefully monitored and deemed safe. Collaboration between different organizations, supported by scientific research and the practice of exercise specialists and trainers, would be advisable so that the newly developed guidelines have a broad context and are easily applicable by pregnant women with different needs.

While moderate-intensity PA is generally recommended for health benefits, some studies suggest that higher intensity activities may be necessary to achieve certain health benefits. There are studies suggesting that moderate-intensity PA may not always be sufficient to achieve certain benefits, and that higher intensity activities may be needed to obtain specific health advantages. For instance, highlighted that while studies show that moderate-intensity exercise is effective in reducing the risk of coronary heart disease, some studies suggest that only heavy or vigorous activity confers benefits (41). This implies that moderate-intensity exercise alone may not provide the same level of benefit as more intense PA. Moreover, Powell’s review (42) concluded that vigorous activity may offer greater cardioprotective benefits than moderate-intensity activity comparing the cardioprotective benefits of vigorous and moderate-intensity aerobic activity. This suggests that higher intensity exercise may be more effective in certain health outcomes compared to moderate-intensity exercise alone. Additionally, Janssen and Ross found that vigorous-intensity PA is related to metabolic syndrome independent of the PA dose, indicating that the intensity of PA plays a significant role in health outcomes (43).

What’s interesting, there are more and more studies supporting the safety and maternal and foetal health benefits of engaging in high-intensity PA during pregnancy. Hinman (44) found that moderate-and high-intensity exercise in normal pregnancies is safe for the developing fetus and has several important benefits.

High-intensity interval training (HIIT) during pregnancy has gained attention as an effective and time-efficient exercise modality (44). Wowdzia et al. proved that a single HIIT session was well tolerated by both the mother and the foetus in terms of both metabolic (45) and cardiorespiratory parameters (46). While the specific effects of long-term HIIT during pregnancy are still being explored, preliminary studies suggest that HIIT can be a suitable exercise option for pregnant women, offering benefits such as improved respiratory and cardiovascular adjustments, enhanced exercise performance, and body composition (47). Additionally, 8-week HIIT programme during pregnancy has been shown to be an effective intervention in improving mothers’ mental health and preventing depression (48, 49). Szumilewicz et al. (50) mentioned that while HIIT is popular among women in general, there’s limited data on its safety and effectiveness during pregnancy. What’s more, the authors highlighted that conservative guidelines from the past discouraged strenuous exercise during pregnancy, but more recent research suggested a need for updated recommendations. They also pointed out that some pregnant women might be interested in continuing HIIT or seeking information online, emphasizing the importance of developing and disseminating reliable guidelines on this topic. Interestingly, only Polish guidelines have so far initiated a discussion on HIIT in pregnancy (33). It should be noted that EBCOG has been quite cautious about high-intensity PA, noting the potential risks (32). Nevertheless, the topic of high-intensity PA in pregnancy should certainly be considered a visible trend, both in scientific research and in the context of planning training programs for the pregnant population.

While exercising at high altitudes during pregnancy can stimulate beneficial physiological adaptations like increased hemoglobin concentrations and improved cardiovascular function, caution is advised due to potential risks. Keyes et al. (51) found that babies born to women who resided at high altitude during pregnancy were more likely to have intrauterine growth retardation (IUGR), need oxygen at birth, and be admitted to the NICU than lowland residents with or without altitude travel during pregnancy. However, in the same study the authors concluded that women who are active in outdoor sports and travel to high altitude have a low rate of complications. Umar et al. (52) observed that pregnant women living at higher altitudes often exhibit increased hemoglobin, enhancing oxygen transport to both mother and fetus, potentially mitigating risks like intrauterine growth restriction. Additionally, regular PA at altitude may improve maternal health outcomes, including reduced incidences of gestational diabetes and hypertensive disorders (51). However, high-altitude environments, particularly above 2,500 meters, can pose risks such as altitude sickness, adversely affecting both maternal and fetal health (51).

Care must be taken in making recommendations regarding exercising at altitude during pregnancy since there is so little supporting evidence and research in this topic (52).” There is no data on the safety of altitude training for competitive female athletes. Therefore, pregnant women should engage in altitude exercise cautiously, ensuring acclimatization and monitoring for signs of distress. They should consult healthcare providers to adapt exercise regimens that ensure safety and optimize health outcomes.

### 4.2 Limitations

Even though efforts were made to include all illegible recommendations in the review, some relevant documents may have been overlooked. Moreover, documents reported in languages other than English or issued by institutions other than government, medical, or sports institutions were excluded.

Because available recommendations regarding exercise during pregnancy not always have the character of scientific work and more often constitute an official representative position of institution, it was in some cases impossible to find them in scientific databases. Nevertheless, the authors tried to adopt such a procedure for searching for material for analysis that would make it effective and repeatable, while being aware that some documents that met the criteria for inclusion in the analysis could have been omitted.

## 5 Conclusion

1. There is a consistent message across guidelines and recommendations: Moderate-intensity PA during pregnancy is generally safe and provides numerous health benefits for both mother and baby. This strengthens the importance of clear and consistent public health messages that encourage pregnant women to engage in regular moderate-intensity PA.
2. While moderate-intensity PA provides a solid basis for a healthy pregnancy, emerging research suggests that higher-intensity exercise may be more effective in achieving specific health outcomes, including improved cardiovascular health and metabolism. This highlights the opportunity for future guidelines to take a more balanced approach, taking into consideration individual factors such as pre-pregnancy fitness levels, desired health outcomes and potential risks, to provide more personalised recommendations.
3. While moderate-intensity PA is generally safe and beneficial, there is a need for more personalised recommendations, particularly for pregnant athletes or those with a history of intense exercise or exercising at higher altitudes. Guidelines should take into account individual predisposition, fitness level and training history to provide tailored advice that optimises both maternal and fetal outcomes.
4. Importantly, we have seen much more open attitudes towards higher-intensity PA during pregnancy than a few years ago. It appears to be a trend that requires scientific recognition and development of recommendations for practitioners working with pregnant population.

## Data Availability

The original contributions presented in the study are included in the article/Supplementary material, further inquiries can be directed to the corresponding author.
